# Estimating spatio-temporal fields through reinforcement learning

**DOI:** 10.3389/frobt.2022.878246

**Published:** 2022-09-05

**Authors:** Paulo Padrao, Jose Fuentes, Leonardo Bobadilla, Ryan N. Smith

**Affiliations:** ^1^ Knight Foundation School of Computing and Information Sciences, Florida International University, Miami, FL, United States; ^2^ Institute for Environment, Florida International University, Miami, FL, United States

**Keywords:** spatio-temporal fields, reinforcement learning, partial differential equations, autonomous navigation, environmental monitoring

## Abstract

Prediction and estimation of phenomena of interest in aquatic environments are challenging since they present complex spatio-temporal dynamics. Over the past few decades, advances in machine learning and data processing contributed to ocean exploration and sampling using autonomous robots. In this work, we formulate a reinforcement learning framework to estimate spatio-temporal fields modeled by partial differential equations. The proposed framework addresses problems of the classic methods regarding the sampling process to determine the path to be used by the agent to collect samples. Simulation results demonstrate the applicability of our approach and show that the error at the end of the learning process is close to the expected error given by the fitting process due to added noise.

## 1 Introduction

The use of autonomous underwater and surface vehicles (AUVs and ASVs) for persistent surveillance in coastal and estuarine environments has been a topic of increasing interest. Examples of studies enabled by these vehicles include the dynamics of physical phenomena, such as ocean fronts, temperature, the onset of harmful algae blooms, salinity profiles, monitoring of seagrass and coral reefs, and fish ecology.

Due to the stochastic nature of these vital environments and the large spatial and temporal scales of significant processes and phenomena, sampling with traditional modalities (e.g., manned boats, buoys) is sparse and predictive models are necessary to augment decision-making to ensure that robotics assets are at the right time and the right place for sampling. However, no single model provides an informed view or representation of these or any other ocean feature that enables intelligent sampling in a principled manner. Therefore, it is critical to forecasting where a robot should sample in the immediate future so that sufficient information is provided on getting to the desired location within a dynamic environment.

**FIGURE 1:**
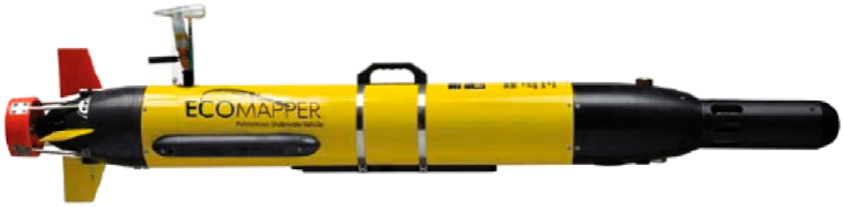
YSI-Ecomapper autonomous underwater vehicle.

Our ideas are inspired by commonly used underwater vehicles in environmental and infrastructure monitoring problems such as the AUV Ecomapper shown in Figure 1. This vehicle can measure water quality parameters, currents, and bathymetric information. However, its mission endurance is limited to a few hours due to its battery constraints, therefore, efficient sampling strategies are needed.

The contributions of this paper are the following:

1) A novel framework combining classic methods with reinforcement learning to estimate ocean features, which are modeled as spatio-temporal fields.
2) A technique to get a set of informative samples to estimate spatio-temporal fields, which an agent can collect and process.
3) An extension to the classical partial differential equations fitting methods to estimate models incorporating reinforcement learning.

This paper is an expansion of our preliminary work in Padrao et al. (2022) and extends it to include the estimation of ocean features using partial differential equations. The rest of the paper is organized as follows. In Section 2, we review related work to our approach. Section 3 gives the preliminaries needed to build our method and formulate our problem. Section 4 presents the Reinforcement Learning Methods used to solve our approach, and the results are presented in Section 5. Finally, Section 6 concludes our paper and gives direction for future work.

## 2 Related work

### 2.1 Oceanic monitoring sampling

Over the last decade, it has become clear that autonomous marine vehicles will revolutionize ocean sampling. Several researchers have investigated approaches for ASVs and AUVs for adaptive ocean sampling Yuh (2000)-Smith et al. (2010c) and fundamental marine sampling techniques for ASVs and AUVs are discussed in Singh et al. (1997). Besides control algorithms for Oceanic Sampling, an alternative approach is to use static sensor placements to maximize information gathering Zhang and Sukhatme (2008).

### 2.2 Adaptive sampling with marine vehicles

Our work connects also with research on control design for AUVs for adaptive ocean sampling, Yoerger and Slotine (1985); Frazzoli et al. (2002); Low et al. (2009); Rudnick and Perry (2003); Yuh (2000); Frank and Jónsson (2003); Graver (2005); Barnett et al. (1996); Carreras et al. (2000); Ridao et al. (2000); Rosenblatt et al. (2002); Turner and Stevenson (1991); Whitcomb et al. (1999, 1998); McGann et al. (2008b), McGann et al. (2008a), McGann et al. (2008c), Yoerger and Slotine (1985)-McGann et al. (2008c). Applications of ocean sampling techniques for autonomous vehicles are discussed in Singh et al. (1997)-Eriksen et al. (2001). This body of research differs from the proposed research in that we plan to utilize predictive models in the form of Partial Differential Equations (PDE) to enable effective sampling, navigation, and localization within dynamic features.

### 2.3 Reinforcement learning in marine robotics

Reinforcement learning in marine robotics, especially model-free methods, is an attractive alternative to finding plans for several reasons. First, executing marine robotics experiments and deployments is expensive, time-consuming, and often risky; controllers learned through RL can represent significant time and cost savings and shorten the time to deployment. Second, system identification can sometimes be challenging in marine environments due to several factors such as unmodeled dynamics and environment’s unknowns; for that reason, model-free RL approaches can be an alternative in these scenarios. Examples of approaches that have used RL for ASVs or AUVs include path planning Yoo and Kim (2016), control Cui et al. (2017) and tracking Martinsen et al. (2020).

### 2.4 Machine learning for partial differential equations

Our ideas are also connected to the use of Machine Learning models in the context of Partial Differential Equations. Due to their usefulness and impact in several domains, there have been efforts to use modern machine learning techniques to solve high dimensional PDEs Han et al. (2018), find appropriate discretizations Han et al. (2018), and control them Farahmand et al. (2017).

## 3 Preliminaries and problem formulation

### 3.1 Partial differential equations

Partial differential equations (PDEs) have been used to model water features of interest such as pH, temperature, turbidity, salinity, and chlorophyll-A. Depending on the nature of their motion, they can be modeled through diffusion, advection or a combination of both. It is important to evaluate how they behave given certain initial conditions to understand their evolution in time. We model the ocean features of interest as a scalar field $f:R^{2}\times \left[0,\infty \right)\to R$ .

#### 3.1.1 Advection equation

The advection equation models how a given ocean feature (e.g., algae bloom, oil spill, chemical contaminants, etc.) is transported by a given flow which goes in the direction of $b\in R^{2}$ ; it is also called the *transport equation*. The model the space is given by 1.

$$\begin{array}{cc} \frac{\partial f}{\partial t}+b\cdot \nabla f & =g\left(x,t\right),\;\text{for}\;\left(x,t\right)\in R^{2}\times \left(0,\infty \right)\\ f\left(x,0\right) & =h\left(x\right),\;\text{for}\;t=0 \end{array}$$ (1)

has the solution shown in Evans (1998).

$$f\left(x,t\right)=h\left(x-tb\right)+\underset{{\text{By the Duhamel}}^{\prime }\text{s principle}}{\underset{︸}{\int_{0}^{t}g\left(x+\left(s-t\right)b,s\right)ds}}.$$ (2)

Provided that $g\left(x,t\right)\in C\left(R^{2}\right)$ and has a compact support for each t∈[0,∞) . This function *g* models if there are sinks or fonts of the ocean feature in the domain. If the sign of *g* (**x**, *t*) is positive, we consider that point as an ocean feature source; if it is negative, we consider it as an ocean feature sink. On the other hand, *h*(**x**) is the initial distribution of the ocean feature at the beginning.

### 3.2 Estimation of the parameters of a PDE

Once we chose a PDE as a model, it is crucial to estimate the parameters of the PDE to get a reliable model. This problem belongs to the family of inverse problems since those parameters are sensitive to the observations and given initial conditions Richard et al. (2019) Antman et al. (2006). Because of this sensitivity, it is computationally expensive to find the PDE parameters. There are optimization-based techniques to solve this problem. These techniques problems balance the fitting parameter to the observations and the model sensitivity to those parameters. One of most used methods is the Tikhonov regularization technique Nair and Roy (2020) Bourgeois and Recoquillay (2018). It comprises solving a regularized optimization problem to get a regularized solution. It can be highly efficient depending on the regularization norm (especially if the *L* ^2^ norm is used). However, it depends on the regularization constant to achieve good results.

Other approaches to solving the PDE estimation problem take advantage of Bayesian theory Xun et al. (2013). In this case, bayesian learning is connected to regularization since the regularization problem coincides with the maximization of the likelihood of the parameters given the observations Bishop (1995). Therefore, Machine Learning techniques have been proposed to take advantage of the capability of the models to discover hidden relationships between the input data and the final estimation Jamili and Dua (2021). Most of those models use the fact that the samples are given in advance. This work proposes a learning mechanism to select samples that can reasonably estimate the model without exploring the complete domain. This principle has been used in numerical integration problems resulting in several quadrature rules, such that Gauss–Kronrod, Gauss-Legendre, or Newton cotes Kincaid et al. (2009). Those methods have proven to be more efficient since they can give reliable estimations using few points. In this work, we employ an intelligent agent capable of sampling the environment, searching for reliable samples, and using them to compute the parameters of a PDE. Also, this allows estimating the ocean feature behavior in the domain according to Eq. 2.

### 3.3 Model definition

We modeled the marine environment as a 2-D water layer (representing, for example, the surface) denoted as $W\subset R^{2}$ where W is an open and bounded set. The obstacle-free state space for our robot is represented by S=W\O , where O represents the set of locations that are not accessible to the robot.

To estimate the flow field, we define a scheme of fitting problems based on the known initial conditions of Eq. 1 *h*(**x**) and the current samples acquired by the agent. First, we expect to collect samples *y*_*i*_ at the location **x**_*i*_ and time *t*_*i*_ for *i* = 1, *…* , *n* such that the field minimizes the mean square error of the collected samples. Taking advantage of the closed solution described in the homogeneous version of Eq. 1, the fitting error function *e*_*f*_(***b***) is defined as

$$e_{f}\left(b;x_{1},\ldots ,x_{n}\right)=\frac{1}{n}\overset{n}{\underset{i=1}{\sum }}{\left(f\left(x_{i},t_{i}\right)-y_{i}\right)}^{2}=\frac{1}{n}\overset{n}{\underset{i=1}{\sum }}{\left(h\left(x_{i}-t_{i}b\right)-y_{i}\right)}^{2}$$ (3)

where *n* is the number of collected samples. Next, we define the fitting error *e*_*f*_ (**x**_1_, *…* , **x**_*n*_) associated to the locations

$$e_{f}\left(x_{1},\ldots ,x_{n}\right)=\underset{b\in R^{2}}{\min}e_{f}\left(b;x_{1},\ldots ,x_{n}\right).$$ (4)

The fitting error expressed in Eq. 4 measures how well can the best fitted model prediction of the given samples (i.e., predict *y*_*i*_ given **x**_*i*_ and a parameter vector ***b***. It is the “best” in the sense that is the minimum achievable error produced by the model given the samples **x**_1_, … , **x**_*n*_). Nevertheless, we can notice that if the locations **x**_1_, *…* , **x**_*n*_ are wrongly chosen, the fitting error can be low, but its capability of estimating the entire field may lead to over-fitting problems. To handle this issue, we add a new error term based on how well one sample can be predicted using the remaining ones. This is known as cross-validation. In this case, we propose the following cross-validation scheme. For each 1 ≤ *i* ≤ *n* let $b_{i}^{*}$ defined as

$$b_{i}^{*}=\underset{b\in R^{2}}{arg\;\min}e_{f}\left(b;x_{1},\ldots ,x_{i-1},x_{i+1},\ldots ,x_{n}\right).$$ (5)

We define the cross validation error *e*_*cv*_ (**x**_1_, *…* , **x**_*n*_) as

$$e_{cv}\left(x_{1},\ldots ,x_{n}\right)=\frac{1}{n}\overset{n}{\underset{i=1}{\sum }}{\left(h\left(x_{i}-t_{i}b_{i}^{*}\right)-y_{i}\right)}^{2},$$ (6)

and it measures on average how well the samples can fit a model, which is estimating the remaining sample. This avoids the over-fitting problems and allows to measure how reliable are the taken samples. Lastly, we define the total error *e*_*total*_ (**x**_1_, *…* , **x**_*n*_) or just *e*_*total*_ as

$$e_{\mathrm{total}}\left(x_{1},\ldots ,x_{n}\right)=e_{f}\left(x_{1},\ldots ,x_{n}\right)+e_{cv}\left(x_{1},\ldots ,x_{n}\right).$$ (7)

This error compound aims to have an equal trade off between the sample estimation measured by *e*_*f*_ (**x**_1_, *…* , **x**_*n*_) and the reliability of the samples measured by *e*_*cv*_ (**x**_1_, *…* , **x**_*n*_).

The agent is modeled as a rigid body that moves in $R^{2}$ and can be described by a non-linear system as

$$\begin{array}{cc} \dot{x} & =f\left(x,u\right)\\ z & =o\left(x,r\right) \end{array}$$ (8)

Such that *f* (**x**, **u**) is the motion model of the vehicle, *o* (**x**, **r**) is the observation model of the vehicle, and **r** are additive, zero-mean noise to account for modeling errors and sensor imperfections.

Let S be the state space, i e., the set of all possible states x∈S and U be the action space, which represents the set of all possible actions. Therefore, a configuration of the vehicle can be described by

$$\begin{array}{cc} x & =\left(x,y,\phi \right)\\ u & =\left(u_{v},u_{\omega }\right) \end{array}$$ (9)

In which (*x*, *y*) is the position of the vehicle and *ϕ* ∈ (−*π*/4, *π*/4) is the vehicle’s heading; the forward speed *v* and the angular velocity of the agent orientation *ω* can be set directly by the action variables *u*_*v*_ and *u*_*ω*_, respectively. The kinematic model of the agent $\dot{x}=f\left(x,u\right)$ is described by Eq. 10.

$$\begin{array}{cc} \dot{x} & =u_{v}\cos\phi +v_{x}\\ \dot{y} & =u_{v}\sin\phi +v_{y}\\ \dot{\phi } & =u_{\omega } \end{array}$$ (10)

where *v*_*x*_ and *v*_*y*_ account for the velocity components of the environment (flow field) in *x* and *y* directions.

Let $x_{S}\in S$ be the initial location of the agent. It is assumed that the agent takes advantage of ocean current dynamics as it drifts and moves forward with or against the currents and rotates clockwise or counterclockwise. Therefore, the action space is defined as

$$U=\left[0,v_{\max}/2,v_{\max}\right]\times \left(\phi_{\min},\phi_{\max}\right)$$ (11)

We discretize the action space to obtain a finite subset of U defined as

$$A=\left\{\left(v_{\max},0\right),\left(v_{\max}/2,0\right),\left(v_{\max},-\phi \right),\left(v_{\max},+\phi \right),\left(v_{\max}/2,-\phi \right),\left(v_{\max}/2,+\phi \right),\left(0,0\right)\right\}$$ (12)

The description of the actions of the agent are summarized in Table 1.

**TABLE 1:** Description of the actions of the agent.

(*v*_max_, 0)	moving forward with maximum speed *v*_max_
(*v*_max_/2, 0)	moving forward at half the speed *v*_max_/2
(*v*_max_, − *ϕ*)	turning clockwise by *ϕ* and moving with maximum speed
(*v*_max_, + *ϕ*)	turning counterclockwise by *ϕ* and moving with maximum speed
(*v*_max_/2, + *ϕ*)	turning clockwise by *ϕ* and moving at half the maximum speed
(*v*_max_/2, + *ϕ*)	turning counterclockwise by *ϕ* and moving at half the maximum speed

For the observation model, we assume that the vehicle uses an IMU to measure its heading angle *ϕ* and has access to GPS at surface level. Also, the vehicle can observe its state with uncertainties due to sensor imperfections and the dynamic nature of the underwater environment. The observation space Z , the set of all possible sensor observations z∈Z , is given by

$$Z=\left(x_{\min},x_{\max}\right)\times \left(y_{\min},y_{\max}\right)\times \left(\phi_{\min},\phi_{\max}\right).$$ (13)

The observation model *o*(**x**) is represented by

$$z=o\left(x,r\right)=Ix+r$$ (14)

Where $r\in R^{3}$ is noise distributed as r∼N(0,Σ) with Σ a diagonal covariance matrix to account for modeling errors and sensor imperfections and *I* is the identity matrix. It was also considered that the measurement noises of each sensor are uncorrelated and have constant covariance.

These elements allow us to formulate the following problem.

**Problem**: *Given an aquatic environment* W *, the action set of the agent* *A* *, the state space* $S=W\times \left(\phi_{\min},\phi_{\max}\right)$ *, the vehicle’s motion model, observations of a given ocean feature in several locations, estimate the flow field (and therefore the ocean feature distribution) by minimizing cross-validation error and error fitting within a given fixed number of steps.*

## 4 Methods

Because of the computational effort required to tackle problems with large state spaces, tabular learning methods may be unfeasible Sutton and Barto (2018). As a result, combining approximation solutions of reinforcement learning methods with generalization techniques yields a computationally viable solution for real-world problems.

To update the agent policy based on actions taken, we suggest using SARSA(*λ*) algorithm in conjunction with a linear function approximation technique based on stochastic semi-gradient descent. The agent is in state $s_{t}\in S$ , takes action *a*_*t*_ ∈ *A*, and receives reward *r*_*t*_ at each time step *t*. In this method, we can estimate the action-value function $\hat{q}\left(s,a\right)$ for the behavior policy *π* in a systematic way. The SARSA(*λ*) algorithm also chooses an action based on the *ɛ*-greedy approach. Therefore, actions with the highest estimated values are chosen with a high probability, but random actions are picked with a low probability *ɛ* independent of their estimated values.

The action-value function approximation is defined as

$$\hat{q}\left(s,a,w\right)\approx q\left(s,a\right)$$ (15)

Where $w\in R^{d}$ is the weight vector of the semi-gradient descent method. The weight vector update is defined by Eq. 16

$$w_{t+1}=w_{t}+\alpha \left[G_{t}-\hat{q}\left(s_{t},a_{t},w_{t}\right)\right]▽\hat{q}\left(s_{t},a_{t},w_{t}\right)$$ (16)

where *α* is the step size, and *G*_*t*_ is the return function. Applying linear function approximation, Eq. 15 can be modified to

$$\hat{q}\left(s,a,w\right)=w^{⊤}x\left(s,a\right)=\overset{d}{\underset{i=1}{\sum }}w_{i}x_{i}\left(s,a\right)$$ (17)

Where $x\in R^{d}$ is the feature vector. Each component *x*_*i*_ (**s**, *a*) of the feature vector corresponds to a feature of the state-action pair (**s**, *a*) and maps it to a real value. As a result, the gradient of the approximate action-value function can be modified as $▽\hat{q}\left(s_{t},a_{t},w_{t}\right)=x\left(s_{t},a_{t}\right)$ and Eq. 16 reduces to

$$w_{t+1}=w_{t}+\alpha \left[G_{t}-\hat{q}\left(s_{t},a_{t},w_{t}\right)\right]x\left(s_{t},a_{t}\right)$$ (18)

### 4.1 Reward function design

In reinforcement learning problems, designing a reward function is not a trivial task, Ng et al. (1999). To avoid spurious exploration, we defined a terminal condition with a fixed number of observations taken to determine when to reset the environment for a new episode. To encourage the agent to minimize the fitting and cross-validation errors within a given number of steps, we provide a reward that is inversely proportional to the sum of the errors at the terminal state. For each episode, the agent collects 20 observations, and the reward function is defined as

$$r\left(s,a\right)=\left\{\begin{array}{cc} \frac{100}{e_{\mathrm{total}}},\; & \text{if the number of observations}=20\\ c_{2},\; & \text{if the number of observations}<5\\ c_{1}c_{2}^{1.5},\; & \text{otherwise} \end{array}\right.$$ (19)

where *c*_1_ is the ratio between total error at previous and current step and $c_{2}=1-{\left(\frac{e_{\mathrm{total}}}{20}\right)}^{0.4}$ .

### 4.2 Linear methods and feature construction: tile coding

**FIGURE 2:**
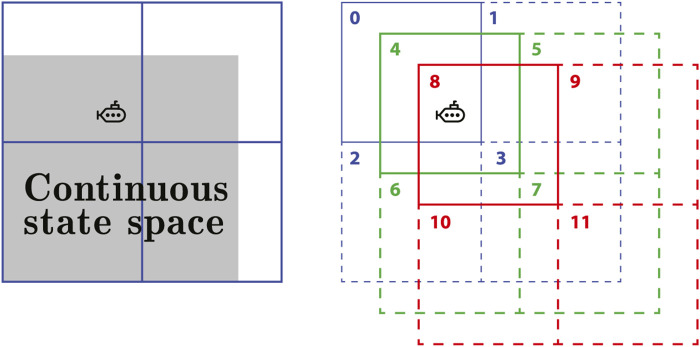
An example of tile coding representation of a continuous 2D state space. The agent is a point in the state space to be represented by the active tiles of the three tilings. Active tiles are described by solid lines and have a value of 1. Inactive tiles are described by dashed lines and have a value of 0. Therefore, the feature vector is **x**(*s*) = [1, 0, 0, 0, 1, 0, 0, 0, 1, 0, 0, 0].

In reinforcement learning systems, feature construction is critical since it values each state of the agent. The main techniques for feature construction of linear methods are polynomial-based, Fourier basis and tile coding Sherstov and Stone (2005). As such, tile coding is a computationally effective feature design technique that divides the state space into divisions called tiles. Each element in the tiling is referred to as a tile. Different tilings are separated by a fixed-size fraction of the tile width Sutton and Barto (2018). If there are *n* tilings and each tiling has *m* × *m* tiles, the feature vector is $x\left(s\right)\in R^{n\times m\times m}$ . One of the main advantages of using tile coding with binary feature vectors is that the weighted sum in the approximate value function (Eq. 17) is easy to compute. Figure 2 shows an example of the representation of tile coding for two-dimensional continuous state space. In this case, **x**(**s**) is a feature vector with twelve components, one for each tile in each tiling. Each component of **x**(**s**) is inactive (zero-valued) except active components *x*_0_(**s**), *x*_4_(**s**) and *x*_8_(**s**) that corresponds to the current location states of the agent. As a consequence, there are *n* active features in **x**(**s**) because every position in state space falls into precisely one tile in each of the *n* tilings. Let the weight vector $w={\left[w_{0},\ldots ,w_{11}\right]}^{⊤}$ and the action space be *A* = {*a*_0_, *a*_1_, *a*_2_}. The feature vector regarding actions *a*_0_, *a*_1_ and *a*_2_ is **x** (**s**, *a*_0_) = **x** (**s**, *a*_1_) = **x** (**s**, *a*_2_) = [1,0,0,0,1,0,0,0,1,0,0,0]^*⊤*^. Thus, the action-value function approximation $\hat{q}\left(s,a,w\right)$ described in Eq. 17 is computed as

$$\hat{q}\left(s,a,w\right)=\overset{d}{\underset{i=1}{\sum }}w_{i}$$ (20)

for each action in action space.

With tile coding, design issues for discrimination and generalization should be taken into account. The number and size of tiles, for example, affect the granularity of state discrimination, or how far the agent must move in state space to change at least one component of the feature vector. Aside from that, the shape of the tilings and the offset distance between them have an impact on generalization. As an example, if tiles are stretched along one dimension in state space, generalization will extend to states along that dimension as well Sutton and Barto (2018).

### 4.3 Eligibility traces in reinforcement learning

In problems with large state spaces, the eligibility trace is a technique to promote computational efficiency of reinforcement learning methods. The eligibility trace is a vector $z_{t}\in R^{d}$ whose components maintain track of which components of the weight vector **w**_*t*_ have contributed to recent state values and temporarily records the occurrence of estimated events. Therefore, components of **w**_*t*_ that most frequently contribute to valuations of previous states are considered *eligible* for an update Singh et al. (1995). Eligibility trace components are updated based on the trace-decay parameter *λ* ∈ [0, 1], which specifies the pace at which the trace fades away exponentially. In contrast with *n*-step methods that perform action-value updates after a given number of steps, eligibility traces provide updates continually over the learning process. For this reason, agent behavior can be modified right after a new state is found rather than being delayed n steps.

The action-value return function *G*_*t*_ is a function approximation of the *n*-step return defined as

$$G_{t:t+n}=r_{t+1}+\cdots +\gamma^{n-1}\hat{q}\left(s_{t+n},a_{t+n},w_{t+n-1}\right),\;t+n<T$$ (21)

where *γ* is the discount rate that regulates the relative importance of near-sighted and far-sighted rewards. Thus, the *λ*-return $G_{t}^{\lambda }$ is written as

$$G_{t}^{\lambda }=\left(1-\lambda \right)\overset{T-t-1}{\underset{n=1}{\sum }}\lambda^{n-1}G_{t:t+n}+\lambda^{T-t-1}G_{t}$$ (22)

In this way, the update rule for the weight vector in Eq. 16 is modified as follows

$$\begin{array}{cc} w_{t+1} & =w_{t}+\alpha \left[G_{t}^{\lambda }-\hat{q}\left(s_{t},a_{t},w_{t}\right)\right]\nabla \hat{q}\left(s_{t},a_{t},w_{t}\right)\\ & =w_{t}+\alpha \delta_{t}z_{t} \end{array}$$ (23)

where the action-value estimation error *δ*_*t*_ is defined as

$$\delta_{t}=r_{t+1}+\gamma \hat{q}\left(s_{t+1},a_{t+1},w_{t}\right)-\hat{q}\left(s_{t},a_{t},w_{t}\right)$$ (24)

The action-value representation of the eligibility trace is defined as

$$\begin{array}{cc} z_{-1} & =0\\ z_{t} & =\gamma \lambda z_{t-1}+▽\hat{q}\left(s_{t},a_{t},w_{t}\right),\;0\leq t\leq T \end{array}$$ (25)

The complete algorithm for SARSA(*λ*) is presented in table 1, Sutton and Barto (2018).

Algorithm 1 SARSA(*λ*) with linear function approximation.

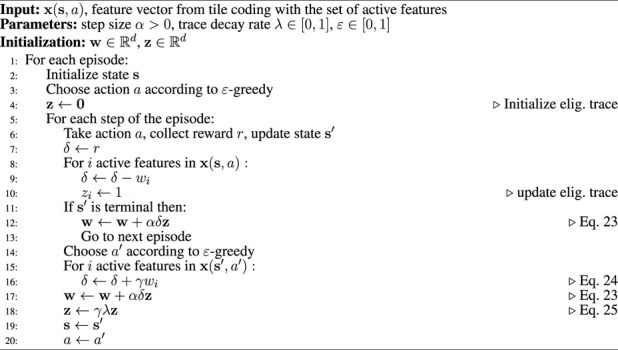

## 5 Results and discussion

**FIGURE 3:**
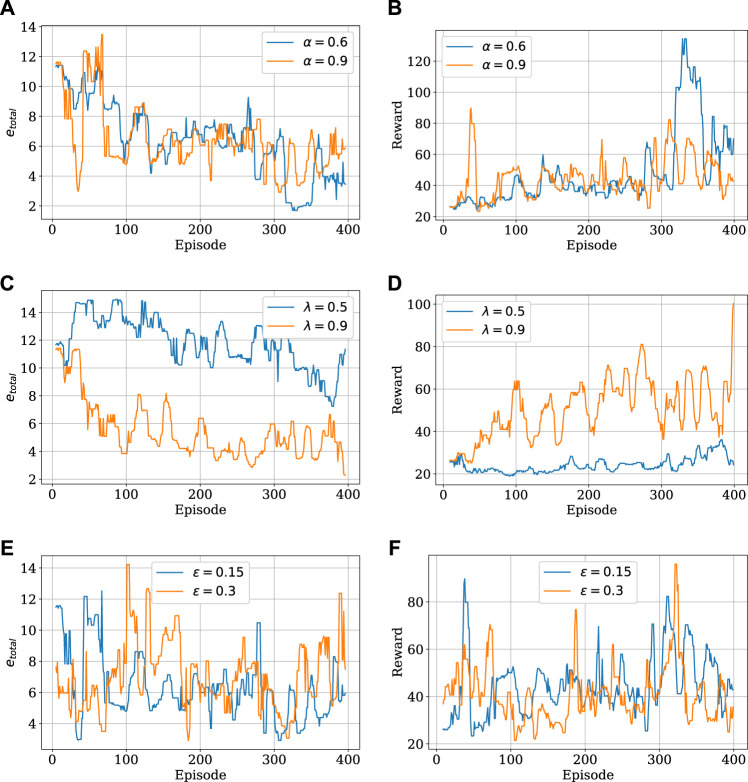
Simulation results of the proposed learning framework with the variation of step size *α* **(A**,**B)**, trace decay rate *λ* **(C**,**D)**, and *ϵ*-greedy parameter **(E**,**F)** with respect to the total number of steps per episode and returns per episode.

Simulation results are presented in Figure 3. For each simulation, we ran a set of simulations consisting of 400 episodes with 20 steps each to investigate how the agent behaves under the effect of the flow field and the variation of the step size *α* and the trace decay rate *λ*. For tile coding, we used eight tilings, each tiling containing 8 × 8 tiles. Thus, the feature vector is $x\left(s\right)\in R^{8\times 8\times 8}$ . Throughout the simulation, the *ɛ*-greedy parameter was fixed at 0.15, indicating that actions with the highest estimated returns are selected 75% of the time. In this way, higher values of the *ɛ*-greedy parameter can lead to an increase in the exploratory behavior of the agent. Besides, to perform the contaminant estimation we selected the functions, keeping the notation at Eq. 1, as *g* (**x**, *t*) = 0 to mean that there are no more sources of the Ocean feature around the domain and

$$h\left(x\right)=a\cdot \exp\left(-\frac{\|x-c{\|}_{q}^{q}}{\sigma^{q}}\right).$$ (26)

*h*(**x**) models the initial distribution of the ocean feature. Where *a* = 100 controls the scale, *q* = 2 manages the decay rate, ‖ ⋅‖_*p*_ is the *L* ^*p*^ norm defined in $R^{2}$ for 1 ≤ *p* ≤ *∞*, *σ* = 40 combined with *q* can be interpreted as the standard deviation of *h*(**x**) and *c* is the point where the ocean feature reaches its maximum. Lastly, each observation was corrupted using Gaussian noise ϵ∼N(0,1) .

Figures 3A,B shows the total estimation error and agent reward with respect to variation of the step size *α*. The step size is interpreted as the fraction of the way the agent moves towards the target. Smaller values of the step size *α* provided an increase in rewards through the episodes and a slight decrease in the estimation error. Additionally, the trace decay rate *λ* was fixed at 0.9. Figures 3C,D shows the total estimation error and agent reward with respect to variation of trace decay rate *λ* of the eligibility trace **z**_*t*_ in Eq. 25. Larger values of *λ* resulted in a significant decrease in the estimation error and an increase in rewards. Figures 3E,F shows the total estimation error and agent reward with respect to variation of the *ɛ*-greedy parameter. Although higher values of the *ɛ*-greedy parameter can lead to higher exploratory agent behavior, simulation shows similar results with different values of *ɛ*.

**FIGURE 4:**
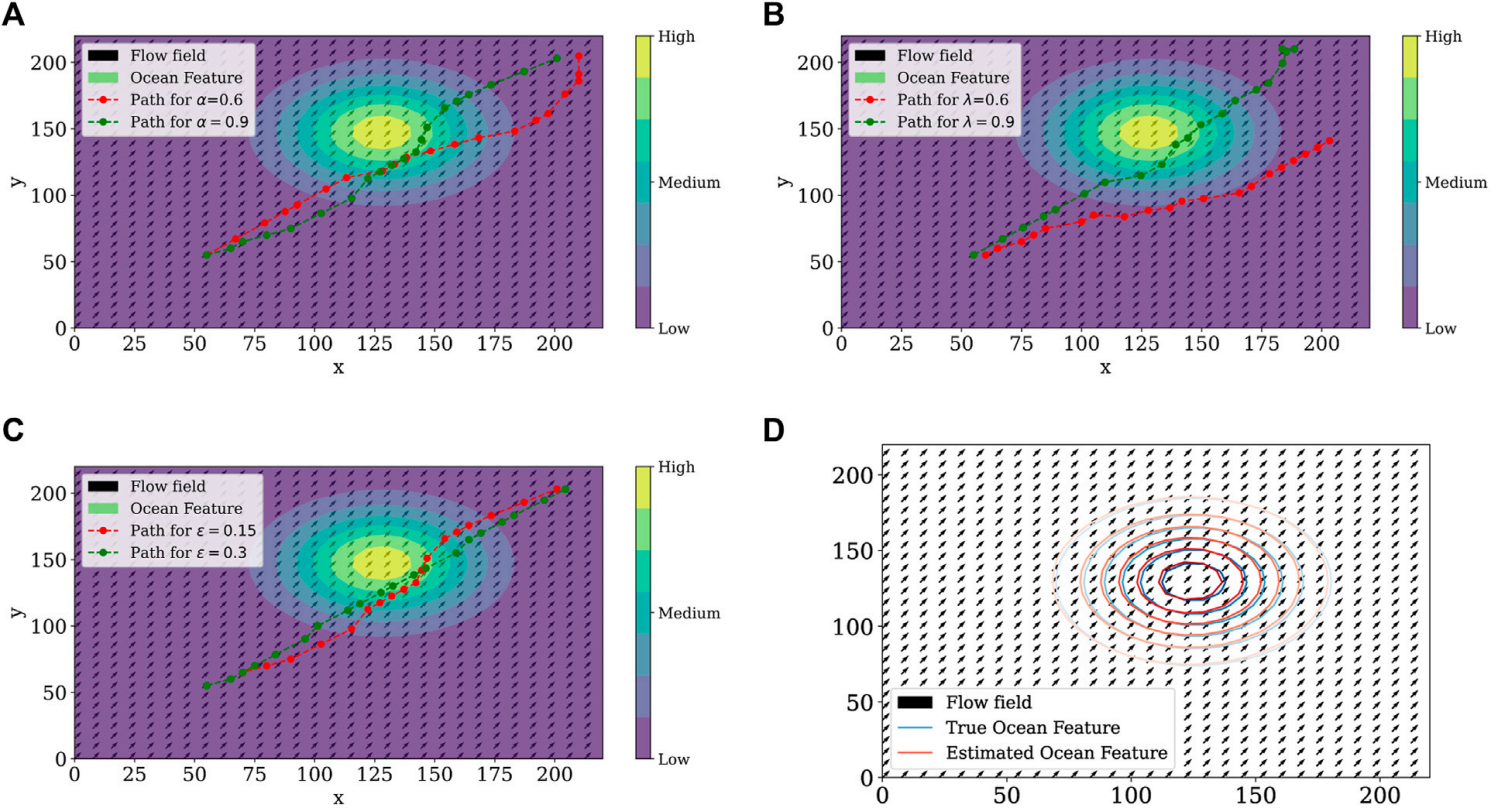
Agent path with the variation of step size *α* **(A)**, trace decay rate *λ* **(B)**, and *ɛ*-greedy parameter **(C)**. **(D)** Difference between the true and the estimated ocean features.

Figure 4 shows different paths taken by the agent in different simulation scenarios. Circles represent level sets of the ocean feature distribution at the end of the simulation. We notice the highest feature concentration location at the center, and the outer circles represent lower ocean feature levels assuming a radial diffusion. Optimal paths have the characteristic of following the ocean feature and crossing its level sets to obtain information at different levels to estimate the entire field.

Finally, Figure 4D shows the difference between the estimated and the true ocean feature distributions at the final time. Both of them are similar once the parameters for the true flow field is **b** = (5,5)^*⊤*^ and the estimated is $\hat{b}={\left(4.928,5.037\right)}^{⊤}$ . We notice that $\|b-\hat{b}{\|}_{2}\approx 0.0809$ , but *e*_*total*_ is close to 2 at the end of the reinforcement learning process. This can be explained because the fitting error *e*_*f*_ is on average the difference between the real observation and the corrupted one. If we assume that the true observations *f* (**x**_*i*_, *t*_*i*_) and the corrupted ones *y*_*i*_ are related by *y*_*i*_ = *f* (**x**_*i*_, *t*_*i*_) + *ϵ*_*i*_ for each *i*, where *ϵ*_*i*_ are i.i.d. Random variables such that $\epsilon_{i}∼N\left(0,\sigma^{2}\right)$ for each *i*. Then, we can notice Bishop (1995) that both, the fitting error *e*_*f*_ and the cross-validation error *e*_*cv*_ approximate the variance *σ* ^2^. Since $E\left[{\epsilon_{i}}^{2}\right]=\mathrm{Var}\left(\epsilon_{i}\right)+E{\left[\epsilon_{i}\right]}^{2}=\sigma^{2}$ and

$$\begin{array}{cc} e_{f}\left(x_{1},\ldots ,x_{n}\right) & =\underset{b\in R^{2}}{\min}\frac{1}{n}\overset{n}{\underset{i=1}{\sum }}{\left(f\left(x_{i},t_{i}\right)-y_{i}\right)}^{2}=\frac{1}{n}\overset{n}{\underset{i=1}{\sum }}{\left(h\left(x_{i}-t_{i}b\right)-y_{i}\right)}^{2}=\underset{\text{as }n\to \infty }{\underset{︸}{\frac{1}{n}\overset{n}{\underset{i=1}{\sum }}\epsilon_{i}^{2}=\sigma^{2}}}\\ e_{cv}\left(x_{1},\ldots ,x_{n}\right) & =\frac{1}{n}\overset{n}{\underset{i=1}{\sum }}{\left(h\left(x_{i}-t_{i}b_{i}^{*}\right)-y_{i}\right)}^{2}=\underset{\text{as }n\to \infty }{\underset{︸}{\frac{1}{n}\overset{n}{\underset{i=1}{\sum }}\epsilon_{i}^{2}=\sigma^{2}}} \end{array}$$ (27)

By the large numbers law. Therefore, *e*_*total*_ = *e*_*cv*_ + *e*_*f*_ ≈ 2.

To increase the complexity of our simulations, we chose to a double-gyre system; a commonly occurring oceanic feature that is relatively easy to model and analyse Provost and Verron (1987), Wolligandt et al. (2020), Smith et al. (2015), Shadden et al. (2005), Shen et al. (1999). The flow is described by the stream-function

$$\psi \left(x,y,t\right)=A\sin\left(\pi f_{dg}\left(x,t\right)\right)\sin\left(\pi y\right)$$ (28)

Where *f*_*dg*_ (*x*, *t*) = *a*(*t*)*x* ^2^ + *b*(*t*)*x*, *a*(*t*) = *μ* sin (*ω*_*dg*_ *t*), *b*(*t*) = 100, −,200*μ* sin (*ω*_*dg*_ *t*) over the domain (0, 200) × (0, 100). In Eq. 28, *A* describes the magnitude of the velocity vectors, *ω*_*dg*_ is the frequency of gyre oscillation, and *μ* is the amplitude of motion of the line separating the gyres, Shadden et al. (2005). Then the flow field produced the double gyre is the vectorial field **v** (*x*, *y*, *t*) = ∇*ψ*(*x*, *y*, *t*).

The PDE (1) considers constant flow fields given by the vector **b**. For this reason, we need to consider an extension of this equation defined in the bounded domain W called the *advection-diffusion* equation

$$\begin{array}{cc} \frac{\partial f}{\partial t}-\rho \Delta f+\nabla \cdot \left(fv\right) & =g\left(x,t\right),\;\text{for}\;\left(x,t\right)\in W\times \left(0,\infty \right)\\ f\left(x,0\right) & =h\left(x\right),\;\text{for}\;t=0\\ \frac{\partial f}{\partial n} & =0,\;\text{for}\;x\in \partial W. \end{array}$$ (29)

Which considers non-constant flow fields, the addition of the diffusion term *ρ*Δ*f* with a small diffusivity coefficient *ρ* and the homogeneous Neumann boundary conditions with outer normal vector **n** is due to the numerical difficulties found and reported when the pure advection equation is solved by numerical methods Evans (1998).

**FIGURE 5:**
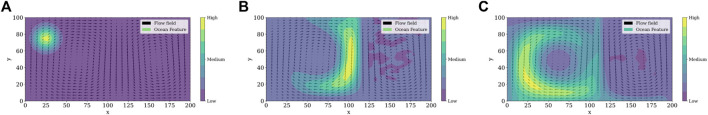
Spread of a given ocean feature through time under the influence of a double-gyre flow field with *A* = 10, *μ* = 0.25, and *ω*_*dg*_ = *π*/5 at **(A)** *t* = 0 s **(B)** *t* = 5 s **(C)** *t* = 10 s.

Figure 5 illustrates the spread of a given ocean feature through time under the influence of a double-gyre flow field.

**FIGURE 6:**
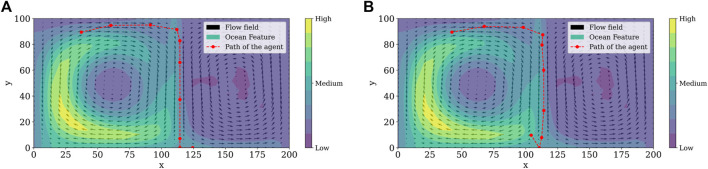
Path followed by the agent, double gyre field to estimate the parameters described in Table 2 at different episodes **(A)** episode 3, **(B)** episode 7.

For the simulation of the reinforcement learning framework and the double-gyre system, we ran a total of 10 episodes with 10 steps each. Although we used tile coding as a computationally effective feature in our reinforcement learning framework, it is still necessary to solve the partial differential equation given in Eq. 29 at each step of each episode. Moreover, to find the fitting and cross-validation errors it is necessary to solve an optimization problem involving the solution of the PDE as a subroutine several times. In order to simulate this computationally intensive optimization algorithm, we took advantage of Florida International University’s Phosphorus, a 20-core Intel(R) Xeon(R) Silver 4114 CPU at 2.20 GHz server, and a Bayesian optimization algorithm intended to handle black box functions which are costly to evaluate. True values for the frequency of gyre oscillation *ω*_*dg*_ and the amplitude of gyre motion *μ* are set to 0.25 and *π*/5 ≈ 0.6283, respectively. Considering only 10 episodes, the learned values for *ω*_*dg*_ and *μ* were 0.2481 and 0.6344, respectively, with the smallest estimation error in episode 7. Learned parameters are summarized in Table 2 and Figure 6 shows the paths taken by the agent at different episodes while estimating the flow field. The agent follows the contaminant, but careful examination should be made at the gyre separation line once the agent could take an undesired action, resulting in feature mistracking. This behavior is illustrated when we compared paths in Figures 6A,B.

**TABLE 2:** Learning simulation parameters and results. True and learned double-gyre model parameters over 10 learning episodes.

Learning simulation parameters	
Number of episodes	10 with 10 steps each
Tile coding	8 tilings with 8 × 8 tiles each
Step size *α*	0.9
Trace decay rate *λ*	0.9
*ɛ*-greedy parameter	0.15
Double-Gyre Model Parameters and Results	
True *μ*	0.25
Learned *μ*	0.2481
True *ω*_*dg*_	*π*/5 ≈ 0.6283
Learned *ω*_*dg*_	0.6344

## 6 Conclusion

In this work, we presented a novel method for estimating a spatio-temporal field using informative samples taken by a trained agent. This allowed estimating the distribution of the ocean feature, keeping track of its localization and distribution at each time. It was possible to address the problem of selecting meaning samples such that they help to perform the estimation of the field. Therefore, this develops a different perspective in estimation procedures, which has been addressed using other techniques having pre-defined models to show *a priori* which samples should be taken.

Moreover, we proposed combining the classical regularization methods used to estimate parameters in partial differential equations with the optimization processes used to carry out those estimates. We merged machine learning techniques, which are more flexible and capable of learning complex patterns from different sources, to choose the sample locations to keep track of and estimate the ocean feature field.

### Future work

For future work, we consider the expansion of the proposed method for 3D environments. This can be accomplished by augmenting the vehicle model (state space, action space, observation space) and validating the proposed framework with deployments in aquatic environments such as in the Biscayne Bay area, Florida, United States. Besides that, it is possible to refine our estimation strategies with cooperative agents. A primary direction for future work is to incorporate a combination of heterogeneous agents in order to provide better estimates of the locations of the ocean feature. In this work, we assume known initial conditions for a given linear, constant flow field. A second direction for future work is to investigate how effective the proposed estimation framework is for time-varying flow fields and actual oceanic data from the Regional Ocean Modeling System (ROMS) Shchepetkin and McWilliams (2005). ROMS data set that provides current velocity prediction data consisting of three spatial dimensions (longitude, latitude, and depth) associated with time. Finally, tracking oceanic features, such as the Lagrangian coherent structures (LCS) contributes to a wide range of applications in ocean exploration Hsieh et al. (2012). Therefore, an additional direction of our work is to expand the current work towards an efficient method for LCS tracking using machine learning techniques.

## Data Availability

The raw data supporting the conclusions of this article will be made available by the authors, without undue reservation.
